# Susac syndrome with prominent dermatological findings and a prompt response to intravenous immunoglobulin, steroids, and rituximab: a case report

**DOI:** 10.1186/s13256-016-0917-4

**Published:** 2016-05-27

**Authors:** Elie Gertner, Michael H. Rosenbloom

**Affiliations:** 1 Section of Rheumatology, Regions Hospital, 640 Jackson Street, St. Paul, MN 55101 USA; 2 Section of Neurology, 401 Phalen Boulevard, Saint Paul, MN 55130 USA

**Keywords:** Susac syndrome, Livedo reticularis, Autoimmune endotheliopathy

## Abstract

**Background:**

Susac syndrome (retinocochleocerebral vasculopathy) is an autoimmune endotheliopathy affecting the precapillary arterioles of the brain, retina, and inner ear. It presents with encephalopathy, branch retinal artery occlusions, and hearing loss. The condition is often under recognized because the clinical symptoms may present at different times and physicians may be unfamiliar with the syndrome. Peripheral findings would be helpful in early diagnosis. There are numerous treatment regimens proposed with varying effectiveness.

**Case presentation:**

We report the case of a 22-year-old Caucasian man in whom there were prominent skin findings, including livedo reticularis and a micropapular eruption which responded promptly to treatment suggesting that skin involvement may facilitate earlier diagnosis. Rituximab has occasionally been used in more refractory disease. We observed a prompt response to the combination of intravenous immunoglobulin, corticosteroids, and rituximab instituted immediately after diagnosis.

**Conclusions:**

A careful search for dermatological manifestations may help with earlier diagnosis. Skin findings may be another marker of endothelial cell involvement. Early use of rituximab as part of the therapeutic regimen may be warranted.

## Background

Susac syndrome (SuS) (retinocochleocerebral vasculopathy) is an autoimmune endotheliopathy affecting the precapillary arterioles of the brain, retina, and inner ear. The major manifestations are the clinical triad of encephalopathy, branch retinal artery occlusions (BRAOs), and hearing loss. First described by neurologist JO Susac in 1979, more than 300 cases have been described to date [1, 2]. The condition is often under recognized because clinical symptoms associated with the triad may present independently at different times and physicians may be unfamiliar with the syndrome. Of note, case reports have described associated dermatological manifestations that might help facilitate earlier diagnosis [3]. Different therapeutic regimens have been described with varying results. More than 10 regimens are listed as options by experts at Cleveland Clinic [4]. While all include intravenous immunoglobulin (IVIG) and steroid therapy, other medications suggested are mycophenolate mofetil, cyclophosphamide, and azathioprine as well as plasmapheresis. The use of rituximab has occasionally been reported in more refractory disease unresponsive to initial therapy [5, 6].

We report a case of SuS, in which there was a prompt response to the combination of IVIG, corticosteroids, and rituximab for the cerebral, ophthalmologic, and otolaryngological manifestations. We further document prominent skin findings including livedo reticularis and a micropapular eruption that responded promptly to treatment, confirming that skin involvement may occasionally be a manifestation of the syndrome and may help with early diagnosis.

## Case presentation

A 22-year-old Caucasian man with a past medical history of depression was admitted to our hospital with a 2-week history of headache, progressive confusion, and behavioral changes, including hallucination. One week prior to his symptoms, our patient developed a livedo-like reticular skin lesion involving both upper extremities and a diffuse pink rash on his chest. A review of his other medical systems was unremarkable. In particular, he had no visual or hearing difficulties and no neck stiffness. There was no infectious exposure and no seizure activity. His medications included sertraline and buspirone.

**Fig. 1:**
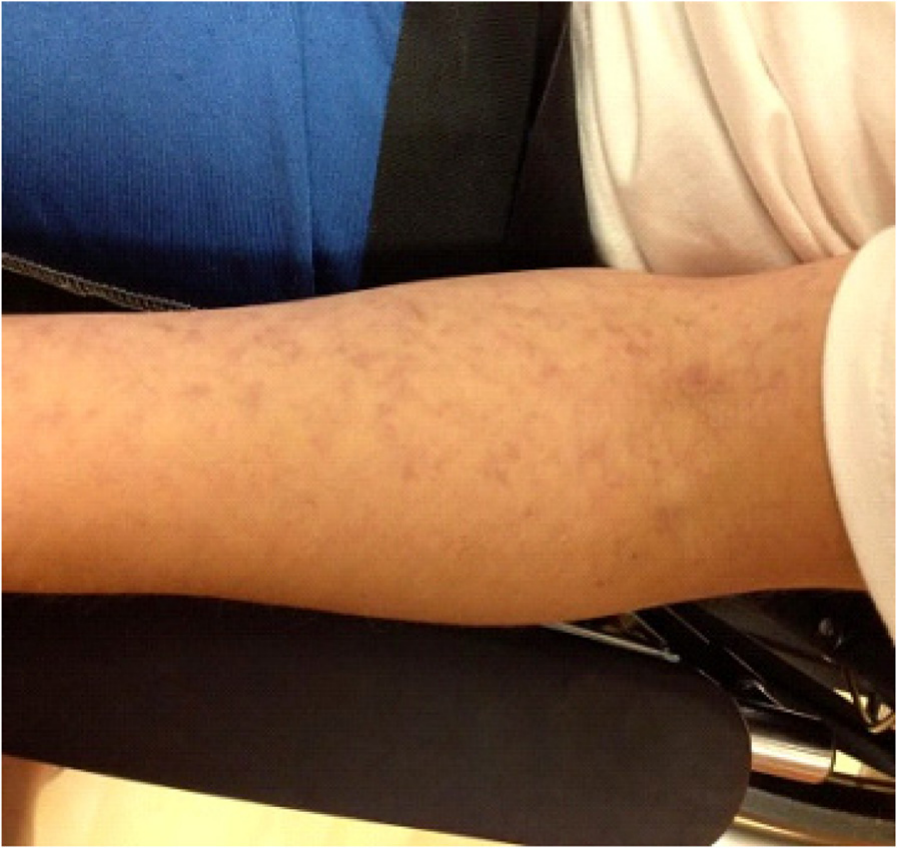
Livedo reticularis-like skin manifestation occurring on both arms, but nowhere else, 1 week before presentation and resolving with treatment

Pertinent findings on examination included a confused young man who was only oriented to person. A dermatological examination revealed skin lesions compatible with livedo reticularis on his upper extremities (Fig. 1) and a diffuse micropapular faint pink eruption with a “sandpaper” quality on his chest and abdomen. There was no prior history of rash. There were no active or effused joints. A neurological examination revealed mild dysmetria with finger-nose-finger testing and limb ataxia. His Mini Mental Status Examination (MMSE) score was 16/30.

Results of a complete blood count, basic metabolic panel, and urine analysis were unremarkable. His results were negative for ANCA. Tests for anti-dsDNA, Sm, Ro, La, centromere, Scl-70, and RNP antibodies were negative, as were all tests for antiphospholipid antibodies and myositis-specific antibodies.

**Fig. 2:**
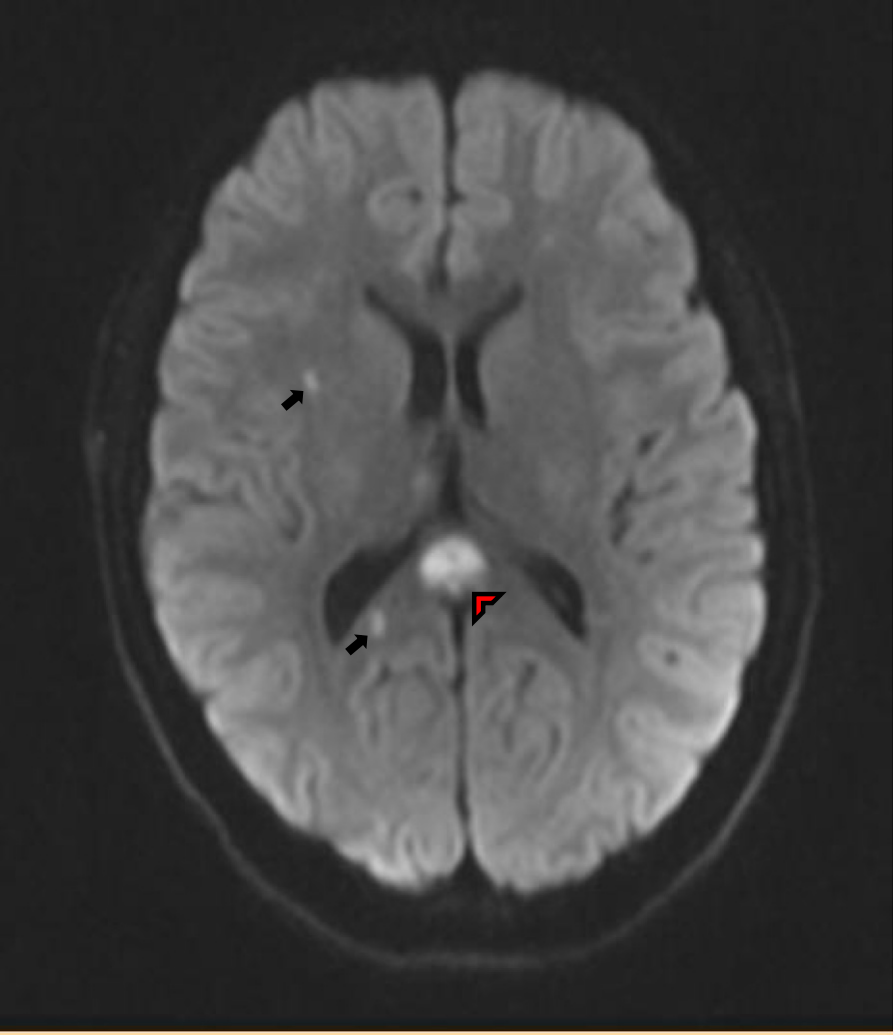
Axial brain magnetic resonance imaging sequences with diffusion-weighted imaging (DWI) revealed multifocal subacute punctate supratentorial white matter lesions (*black arrows*) with the most prominent lesion affecting the posterior corpus callosum (*red arrowhead*). The study also showed T2/fluid attenuated inversion recovery (FLAIR) hyperintensities involving the deep gray nuclei as well as leptomeningeal enhancement (not shown)

Brain MRI with intravenous contrast revealed restricted diffusion involving the splenium of the corpus callosum (red arrowhead) and punctate lesions within the subcortical white matter (black arrows) (Fig. 2). There was enhancement of the leptomeninges as well as the subcortical lesions (not shown). Neither an MRA of his head/neck nor a conventional cerebral angiogram showed any vasculopathy. A lumbar puncture revealed a cerebrospinal fluid (CSF) protein concentration of 278 mg/dl with a normal glucose concentration and cell count. All CSF infectious studies were negative. An EEG showed diffuse slowing consistent with diffuse encephalopathy.

**Fig. 3:**
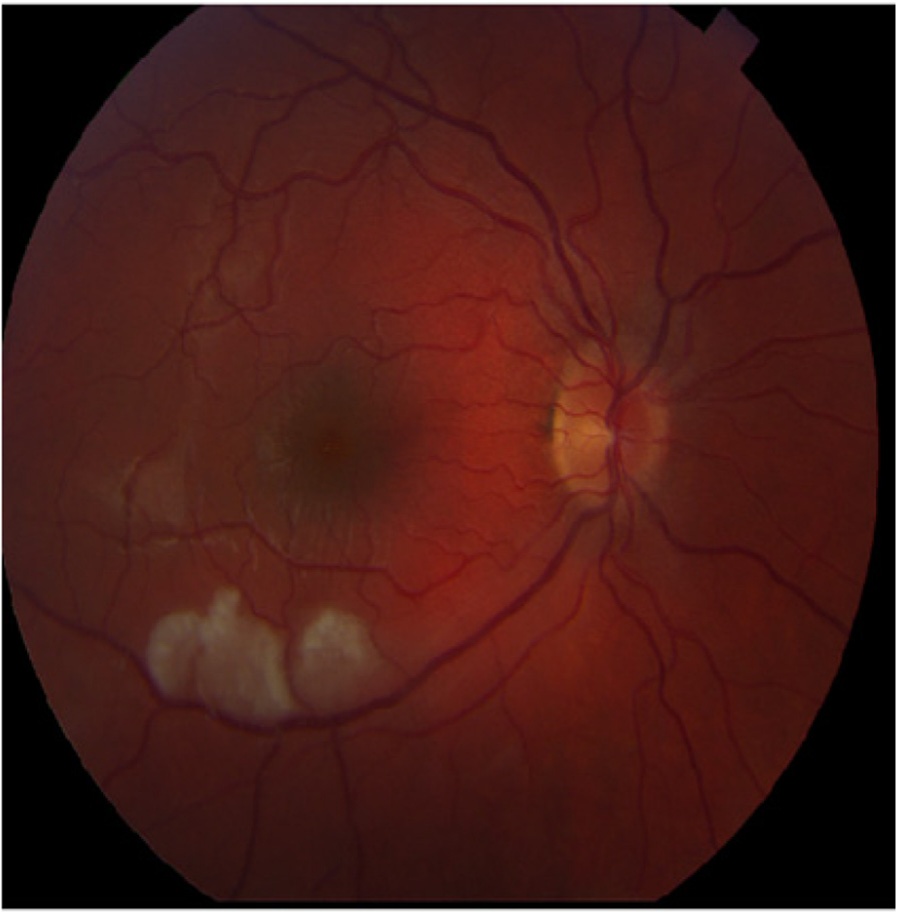
Fundoscopic examination revealed fluffy white patches along the inferotemporal arcade

Fundoscopic examination demonstrated fluffy white patches along the inferotemporal arcade (Fig. 3). Fluorescein angiography confirmed 14 BRAOs and arterial wall hyperfluorescence with leakage of dye.

An audiogram was initially normal and speech recognition thresholds were normal bilaterally.

The livedo lesion was biopsied and examination of the specimen revealed congestion of dermal vessels without inflammation of the vessel walls. The micropapular eruption revealed superficial perivascular lymphocytic infiltration.

Based on the cerebral and eye findings, a diagnosis of likely SuS was made. Our patient was started on methylprednisolone 1 g daily × 5 days given intravenously. On day 1 of the diagnosis he also received IVIG 500 mg/kg. On day 2 he received 1000 mg of rituximab, followed by IVIG 500 mg/kg on day 3 and day 4. He was started on *Pneumocystis jirovecii* pneumonia prophylaxis and a proton-pump inhibitor. On day 6, his steroid was changed to prednisone 1 mg/kg/day given orally. Within a week his confusion and neurological symptoms improved and by the second week his repeat MMSE score was 30/30. A Repeatable Battery for Neuropsychological Status (RBANS) showed cognitive performances ranging from average to superior. The livedo lesions and the micropapular rash resolved within 10 days of treatment.

After 1 week he developed sudden hearing loss. An audiogram showed severe left-sided sensorineural hearing loss. Speech reception thresholds were obtained at 15 decibels hearing level (dBHL) on his right and 50 dBHL on his left. Word recognition was 100 % at 55 dBHL on his right and 8 % at 90 dBHL on his left. An intratympanic steroid injection was performed in his left middle ear and repeated three times over a 2-week period with moderate improvement in his word recognition score.

Repeat fluorescein angiography after 2 weeks and on subsequent testing showed stable filling defects but no new retinal vascular lesions. His left ear word recognition improved over the next month to 52 %.

His treatment regimen over the next 6 months included a gradual taper and discontinuation of steroids, addition of azathioprine as a long-term immunosuppressive agent (mycophenolate mofetil was not tolerated), low-dose aspirin, and IVIG every 2 weeks. Repeat brain MRI showed no progression of his disease and improvement compared with previous studies.

After the first month of treatment, he was able to resume all his daily activities without difficulty or recurrence.

## Discussion

SuS (retinocochleocerebral vasculopathy) is an autoimmune endotheliopathy associated with the clinical triad of CNS dysfunction, BRAOs, and sensorineural hearing loss [2, 7]. Most cases present in patients between 16 and 40 years of age. While CNS symptoms are the most common manifestation, the characteristic clinical triad occurs at disease onset in only a minority of patients. Because of this, the diagnosis may be delayed; in fact, diagnosing SuS solely on the basis of the presence of the complete triad may not be appropriate. CNS symptoms include headaches, often resembling migraine-like headaches, confusion, cognitive impairment, ataxia, and vertigo. Ophthalmological involvement includes many types of disturbances including blurring and flashing. Hearing loss is the major otological manifestation. Other manifestations include myalgia and arthralgia. A few patients have been described to have skin lesions [2, 3].

Involvement of the corpus callosum with evidence of “snowball” lesions is a characteristic MRI finding [8]. Supratentorial white matter and gray matter lesions as well as infratentorial lesions and leptomeningeal enhancement are all found. Fundoscopic examination followed by fluorescein angiography reveals BRAO and arterial wall hyperfluorescence. Audiometry reveals sensorineural hearing loss, which is often bilateral. In patients with suspected SuS, MRI of the brain, fluorescein angiography, and audiometry must be routinely performed even in the absence of clinical manifestations. No specific serological antibody studies have been identified with this syndrome. Cerebrospinal fluid analysis often reveals an elevation of protein, sometimes in combination with a mild lymphocytic pleocytosis. Cerebral angiography is usually normal. A brain biopsy can show microinfarcts and a microangiopathic process with arteriolar wall thickening and lymphocytic infiltration [9].

The differential diagnosis of SuS includes various neurological, psychiatric, and ophthalmologic disorders including multiple sclerosis, vasculitis, infection, neuromyelitis optica, and acute disseminated encephalomyelitis [2, 10].

While the etiology remains unclear, it has been suggested that the microvascular occlusions are mediated by an autoimmune response causing microvascular injury. Anti-endothelial cell antibodies have been detected in some patients. The finding of antiphospholipid antibodies in one case of SuS raised the possibility of an autoimmune endotheliopathy/coagulopathy [1, 11].

Controlled trials of treatment are not available. Expert recommendations suggest treatment regimens similar to those successful with juvenile dermatomyositis, which has some characteristics in common with SuS [12]. More than 10 therapeutic regimens are listed as options by the experts (Robert Rennebohm, MD) at Cleveland Clinic [4]. All include IVIG and steroid therapy. Other medications include mycophenolate mofetil, cyclophosphamide, and azathioprine. Cyclophosphamide for the acute phase of treatment followed by maintenance therapy with methotrexate or long-term therapy with mycophenolate mofetil and methotrexate have been reported to be successful in case reports [13, 14]. The use of rituximab has occasionally been reported in more refractory disease [5, 6]. The ideal regimen has yet to be determined.

The choice of therapies and their mechanism of action are based on the similarities between SuS and juvenile dermatomyositis. The histopathology and electron microscopic findings in the microvasculature of the brain and muscle of patients with SuS are reminiscent of the microvascular abnormalities seen in the muscle and skin of children with dermatomyositis, including endothelial cell swelling, endothelial cell degeneration and necrosis, capillary network destruction, and perivascular lymphocytic infiltration. Because both may represent microvascular endotheliopathies, immunosuppressive medication used in juvenile dermatomyositis may be helpful in SuS [12].

Our patient had remarkable improvement of his CNS function (MMSE score 30/30) and no further eye lesions 2 weeks after the prompt initiation of high-dose corticosteroids, IVIG, and rituximab. One week into the course he developed hearing loss, which was treated with the addition of intratympanic steroids with moderate improvement. The use of intratympanic steroids has been reported on occasion [15, 16]. Although it appears to have been moderately successful in our patient, it remains unclear whether the improvement was related to this local treatment, systemic therapy, or both. Our experience with this patient demonstrates that the majority of patients need immediate, aggressive immunotherapy even before confirmation of the clinical triad of symptoms and that they can respond well, particularly if symptoms have not been present for a long time. Steroids, IVIG, and rituximab may be a useful induction therapy. Steroids have been tapered successfully in our patient and he is being maintained on IVIG infusions and azathioprine.

In this case, livedo reticularis was a prominent manifestation prior to presentation and resolved within 1–2 weeks of treatment. Similarly, the micropapular rash with the nonspecific findings of perivascular lymphocytic infiltration resolved with the treatment rapidly. Dorr *et al*. [2] found 13 reported cases of dermatological findings (mainly livedo reticularis) in SuS. Livedo reticularis results from an impairment of blood flow in the dermal arteries, which is due to many causes including spasm, inflammation, and vascular obstruction. Its appearance at the onset of the disease and resolution with treatment of the underlying disease suggests that the underlying etiological processes responsible for the other manifestations contributed to the livedo reticularis lesions. Turc *et al*. [3] reported a case of SuS preceded by a 2-week history of livedo racemosa. Skin biopsies revealed several occluded dermal arterioles, endothelial cell swelling, and a mild perivascular lymphocytic infiltrate. The pathology in our case was somewhat similar although the dermal arterioles were not as involved. His rashes responded quickly to therapy. Subtle skin lesions may thus be an early and visible manifestation of the syndrome, which otherwise centers around the brain, retina, and inner ear.

## Conclusions

SuS can be a challenging disorder to recognize initially. The characteristic clinical features with appropriate testing can help make the diagnosis. This case report supports an aggressive therapeutic approach involving the combination of steroids, IVIG, and rituximab administered early in the disease process and highlights the associated dermatological manifestations, which may further serve as a marker of endothelial cell involvement.
